# Molecular Characterization of Viral Responsive Protein 15 and Its Possible Role in Nuclear Export of Virus in Black Tiger Shrimp *Penaeus monodon*

**DOI:** 10.1038/s41598-017-06653-7

**Published:** 2017-07-26

**Authors:** Krisadaporn Jaturontakul, Thapanan Jatuyosporn, Pasunee Laohawutthichai, Sun-Yong Kim, Tomoyuki Mori, Premruethai Supungul, Toshio Hakoshima, Anchalee Tassanakajon, Kuakarun Krusong

**Affiliations:** 1 Center of Excellence for Molecular Biology and Genomics of Shrimp, Department of Biochemistry, Faculty of Science, Chulalongkorn University, Bangkok, 10330 Thailand; 2 Structural Biology Laboratory, Nara Institute of Science and Technology, 8916-5 Takayama, Ikoma, Nara 630-0192 Japan; 3 National Center for Genetic Engineering and Biotechnology (BIOTEC), National Science and Technology Development Agency (NSTDA), Pathumthani, 12120 Thailand

## Abstract

A viral responsive protein 15 from *Penaeus monodon* (*Pm*VRP15) has been reported to be important for white spot syndrome virus (WSSV) infection *in vivo*. This work aims to characterize *Pm*VRP15 and investigate its possible role in nuclear import/export of the virus. Circular dichroism spectra showed that *Pm*VRP15 contains high helical contents (82%). Analytical ultracentrifugation suggested that *Pm*VRP15 could possibly form oligomers in solution. A subcellular fractionation study showed that *Pm*VRP15 was found in heavy and light membrane fractions, indicating that *Pm*VRP15 may be associated with endoplasmic reticulum. Double-stranded RNAi-mediated knockdown of *Pm*VRP15 gene expression *in vitro* showed no effect on WSSV copy number in whole hemocyte cells. However, *Pm*VRP15 silencing resulted in an accumulation of WSSV DNA in the nucleus of *Pm*VRP15-silenced hemocytes. Immunofluorescence confocal microscopy showed that *Pm*VRP15 knockdown hemocytes had a much lower level of VP28 (WSSV envelope protein), in comparison to that in the control. It is likely that *Pm*VRP15 may play a role in viral nuclear egress.

## Introduction

White spot syndrome virus (WSSV) is highly infectious and can cause 100% cumulative mortality of farmed shrimps within 3–10 days^1^. In the past decades, shrimp aquaculture industry has been threatened by WSSV, and so far there is no effective treatment for WSSV infection. Understanding the molecular mechanism of WSSV infection will certainly promote the development of potent agents acting against the virus.

WSSV is a bacilliform, non-occluded enveloped virus with the tightly packed nucleocapsid located inside its lipidic, trilaminar membranous envelope^2–5^. The virus replicates and assembles in the host nucleus^6^; and at the late stage of infection, the infected cells are lysed, causing extensive tissue necrosis.

Despite intensive investigations of WSSV infection, the mechanisms of WSSV entry and propagation in shrimp have not yet been fully understood. Several methods including expressed sequenced tag (EST)^7–9^, DNA microarray^10–14^ and proteomic^15–19^, have been used to analyse molecular changes during WSSV infection.

Suppression subtractive hybridization (SSH) of WSSV-challenged *P*. *monodon* hemocytes identified the novel viral responsive protein 15 (*Pm*VRP15) as one of the most highly up-regulated genes in the acute phase of WSSV infection^20^. Tissue distribution analysis showed that *Pm*VRP15 transcript was mainly expressed in the hemocytes of shrimp, and found in all three types of hemocyte (hyaline, semigranular and granular cells). *Pm*VRP15 knockdown resulted in a significant decrease in viral gene expression and cumulative mortality rate of WSSV-infected shrimp, indicating that *Pm*VRP15 is crucial for WSSV propagation. *Pm*VRP15 was also named as *Pm*ERP15, an endoplasmic reticulum (ER) stress-induced protein, which was reported to be important for the survival of WSSV infected shrimps^21^. Recently, WSV399 viral tegument protein was identified as *Pm*VRP15 binder^22^.

This study examines the molecular characteristics of *Pm*VRP15, a 137-amino acid protein containing a putative transmembrane helix, using biophysical techniques including mass spectrometry, circular dichroism, analytical ultracentrifugation and size-exclusion chromatography. In addition, a possible role of *Pm*VRP15 in nuclear import/export has been investigated by measuring WSSV copy number in nuclear and cytoplasmic fractions.

## Results

### Expression and purification of recombinant *Pm*VRP15

Recombinant *Pm*VRP15 was expressed in *E*. *coli* C43 (DE3) and purified by Ni-NTA Sepharose^TM^ 6 Fast Flow and HiTrap SP Fast Flow columns. A major protein band appeared just below 15 kDa, close to the estimated size of r*Pm*VRP15 (15.86 kDa) (See Supplementary Information, Fig. S1). This protein band was confirmed as r*Pm*VRP15 by Western immunoblotting using anti-His monoclonal antibody.

### Molecular characterization of recombinant *Pm*VRP15

**Figure 1:**
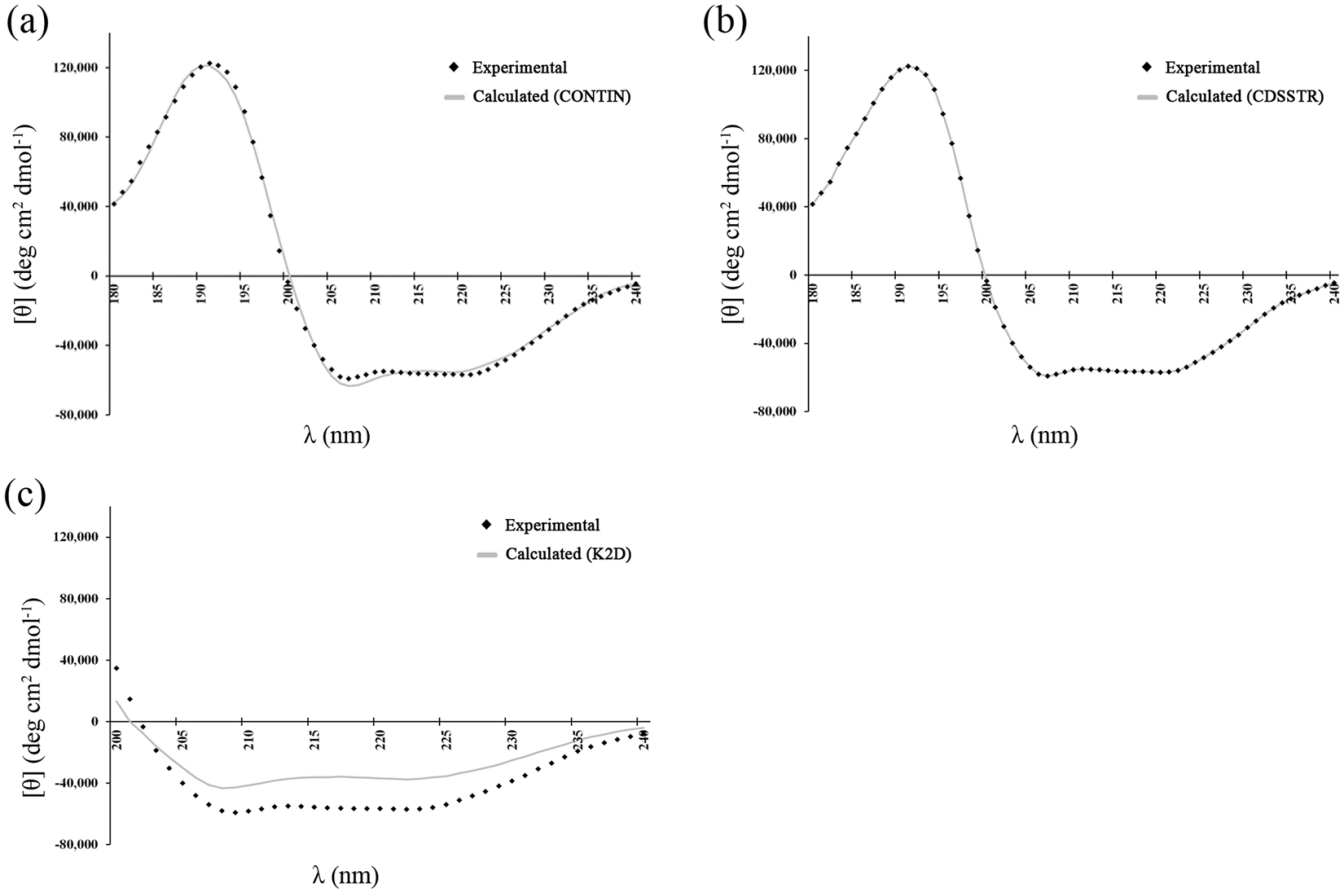
CD spectra of r*Pm*VRP15 analysed by CONTINLL (**a**), CDSSTR (**b**) and K2D (**c**) algorithms via DichroWeb. SMP180 was used as reference dataset. CDSSTR (the best fit) gave calculated secondary structure of 82% α-helix, 4% β-strand, 8% turn and 6% unordered.

MALDI-TOF MS analysis showed that the molecular mass of recombinant *Pm*VRP15 was 15,899.9 Da (data not shown), which corresponded to the calculated MW of *Pm*VRP15 protein (15,859.5 Da), using ExPASy server^23^. CD spectra were analysed by K2D, CONTINLL and CDSSTR algorithms via DichroWeb, using a mixed soluble and membrane protein dataset, SMP180, as reference. CDSSTR produced the best fit to the experimental data (NRMSD = 0.001), in comparison to CONTINLL (NRMSD = 0.044) and K2D (NRMSD = 0.336) (Fig. 1). Output from the CDSSTR/SMP180 suggested that r*Pm*VRP15 is an α-helical protein containing 82% α-helix, 4% β-strand, 8% turn and 6% unordered.

**Figure 2:**
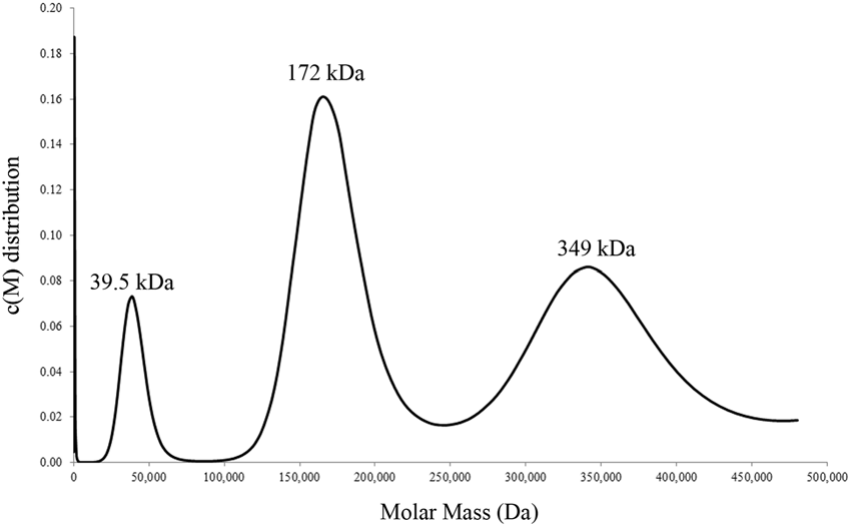
Sedimentation velocity analysis of r*Pm*VRP15. Three peaks of apparent molecular mass of 39.5, 172 and 349 kDa are displayed. The major peak is found at 172 kDa.

Ultracentrifuge sedimentation-velocity analysis of purified r*Pm*VRP15 showed a principal peak at 172 kDa (Fig. 2). Assuming that the molar mass of a single molecule of r*Pm*VRP15 bound to detergent was 39.5 kDa (the first peak in Fig. 2), a major peak of 172 kDa corresponds to a tetramer and that of 349 kDa to an octamer. It is also possible that the first peak is a dimer and the other two are octamer and 16-mer.

**Figure 3:**
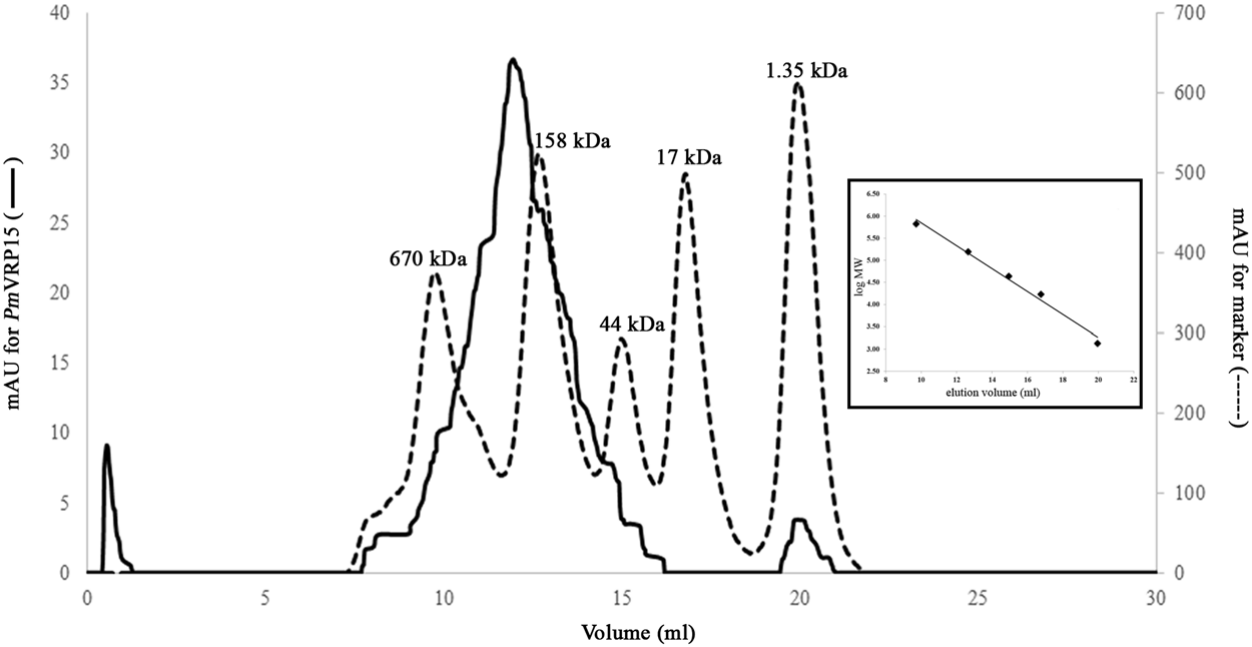
Analytical gel filtration profile of r*Pm*VRP15. Elution profiles of a protein standard mixture and r*Pm*VRP15 are shown in dashed (----) and solid lines (—), respectively. The inset shows a linear relationship between log MW and elution volume.

Size-exclusion chromatography (SEC) was performed to monitor protein aggregation by Superdex 200 column (GE Healthcare). r*Pm*VRP15 was aggregated in equilibration buffer without DM as the protein eluted in early fractions (data not shown). In contrast, r*Pm*VRP15 seemed to be soluble in equilibration buffer with DM and eluted with an approximate MW of ~200 kDa (Fig. 3), which is consistent with the AUC result. This suggested that it is necessary to include detergent in buffer to maintain r*Pm*VRP15 solubility.

### Protein localization of *Pm*VRP15 by subcellular protein fractionation

**Figure 4:**
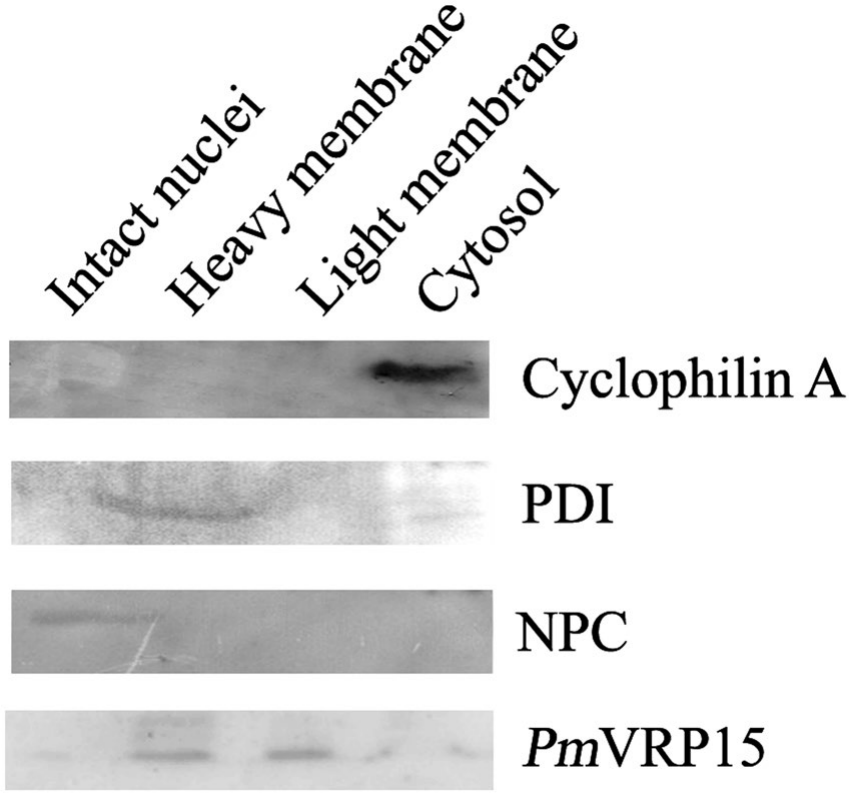
Western blotting analysis of subcellular fractionated WSSV-infected hemocytes. Anti-Cyclophilin A, PDI and NPC antibodies were used as cytosol, ER and nuclear markers, respectively. The blots shown here are cropped from full-length blots in the Supplementary Information.

Intact nuclei, heavy membrane, light membrane and cytosol fractions were extracted from WSSV-infected hemocyte cultures by differential centrifugation. Immunoblot analysis of all fractions using anti-*Pm*VRP15 antibody revealed that *Pm*VRP15 exists in heavy membrane (plasma membrane and rough ER) and light membrane (polysomes, golgi apparatus, smooth ER) fractions (Fig. 4), indicating that *Pm*VRP15 may be an ER-localized protein.

### *Pm*VRP15 mRNA expression in shrimp primary hemocyte culture in response to WSSV

**Figure 5:**
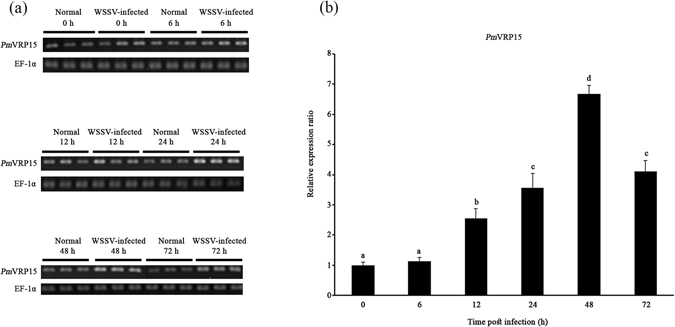
*In vitro Pm*VRP15 mRNA levels in response to WSSV infection. Relative expression ratios of *Pm*VRP15 transcript levels were determined in WSSV-infected *P*. *monodon* hemocytes, compared to control (non-infected) shrimp and standardized against elongation factor-1 alpha (EF-1α) as an internal reference, at 0, 6, 12, 24, 48 and 72 hpi by (**a**) RT-PCR and (**b**) real time RT-PCR. The statistical significance of the data was evaluated using one-way ANOVA followed by post hoc test (Duncan’s new multiple range test). Data differences were considered significant at *P* < 0.01. The gels shown here (**a**) are cropped from full-length gels, included in the Supplementary Information.

*Pm*VRP15 expression in unchallenged and WSSV-challenged *P*. *monodon* primary hemocyte cultures was examined by RT-PCR. Clearly, *Pm*VRP15 transcripts were increased after 12 h post-WSSV infection onwards (Fig. 5a). Real-time RT-PCR confirmed that *Pm*VRP15 mRNA expression was up-regulated by 2.6-, 3.6-, 6.7- and 4.1- fold at 12, 24, 48 and 72 h post-WSSV infection, respectively (Fig. 5b). These results indicated that *Pm*VRP15 was highly expressed in *P*. *monodon* primary hemocyte cultures in response to WSSV infection.

### Double strand RNAi-mediated silencing of *Pm*VRP15 resulted in an increase of WSSV copy number ratio in nucleus/cytoplasm

**Figure 6:**
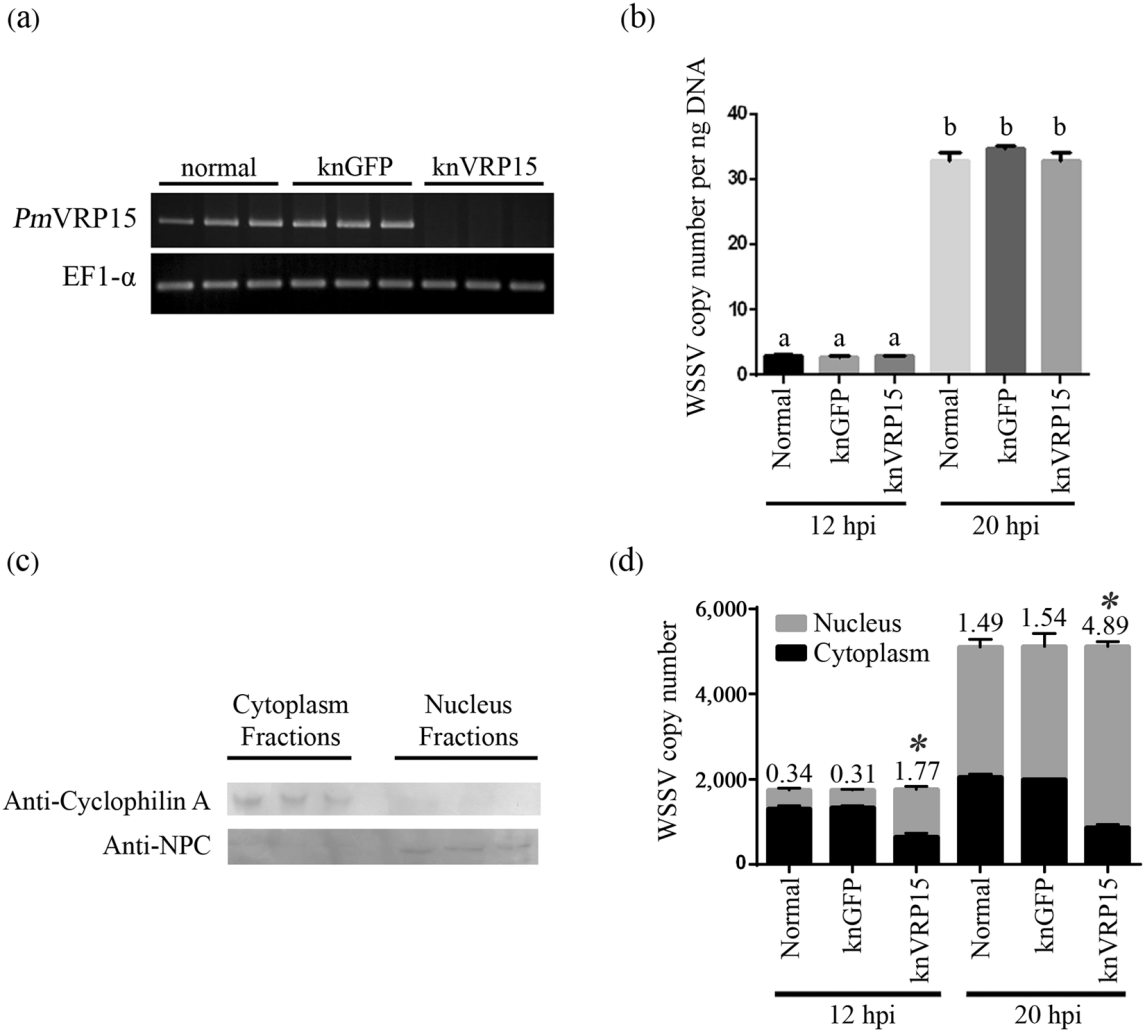
Determination of WSSV copy number in WSSV-infected *P*. *monodon* hemocytes after *Pm*VRP15-silencing. (**a**) RT-PCR analysis of *Pm*VRP15 transcripts in shrimp hemocytes treated with either NaCl, GFP dsRNA or *Pm*VRP15 at 24 hpi. (**b**) WSSV copy number in WSSV-infected whole hemocytes cells at 12 and 20 hpi. (**c**) Western blotting analysis of cytoplasmic and nuclear fractions of WSSV-infected hemocytes. Anti-Cyclophilin A and NPC antibodies were used as cytosol and nuclear markers, respectively. Western blot detection was carried out using Fast Western Blot Kit, SuperSignal^®^ West Femto kit. (**d**) WSSV copy number in cytoplasmic and nuclear fractions of WSSV-infected hemocytes cells at 12 and 20 hpi. The statistical significance of the data was evaluated using one-way ANOVA followed by post hoc test (Duncan’s new multiple range test). Data differences were considered significant at *P* < 0.05. The gel (**a**) and the blot (**b**) are cropped from full-length images, shown in the Supplementary Information.

In this study, RNA interference (RNAi) technique was used to knockdown *Pm*VRP15 transcript in order to investigate the function of *Pm*VRP15. Figure 6a showed that *Pm*VRP15 dsRNA treatment could silence *Pm*VRP15 expression at 24 h-post incubation. In contrast, addition of either GFP or NaCl into hemocyte cultures did not affect *Pm*VRP15 transcript levels. Primary hemocyte cell cultures were then incubated with either *Pm*VRP15 dsRNA, GFP dsRNA or NaCl prior to WSSV infection; and WSSV copy numbers in whole cells were determined at 12 and 20 hpi. Clearly, WSSV copy numbers of hemocytes treated with either *Pm*VRP15 dsRNA, GFP dsRNA or NaCl at 20 hpi were significantly increased, in comparison to WSSV copy numbers at 12 hpi (Fig. 6b). This suggested that WSSV copy number was multiplied in all groups. Interestingly, *Pm*VRP15-silenicng did not change WSSV copy number in knVRP15 group, compared with those in NaCl-treated and knGFP groups.

In a further study, the hemocytes of *Pm*VRP15 dsRNA, GFP dsRNA or the NaCl treated group were collected at 12 and 20 hpi and fractionated into cytoplasmic and nuclear fractions for quantification of WSSV copy number. Western blotting was carried out, in order to confirm that there was no cross contamination between cytoplasmic and nuclear fractions. Anti-cyclophilin A and anti-NPC antibodies were used as subcellular markers for the cytoplasmic and nuclear fractions, respectively. As shown in Fig. 6c, cyclophilin A was detected in the cytoplasmic fractions only and NPC was present solely in the nuclear fractions. This indicated that the cytoplasmic and nuclear fractions were separated well. Thus, WSSV copy numbers in these two fractions were quantified by Real-time PCR.

Figure 6d showed that *Pm*VPR15-knockdown hemocytes contained WSSV copy number ratio in nucleus and cytoplasm of 1.77 at 12 hpi, while NaCl and GFP treated hemocytes showed WSSV copy number ratio in nucleus and cytoplasm of 0.34 and 0.31, respectively. In addition, WSSV copy number ratio in nucleus and cytoplasm of knVPR15 group was 4.89 at 20 hpi, which was much higher than those in both control groups (1.49 and 1.54). These results demonstrated that although control and knVRP groups contained similar amounts of WSSV copy number, it is obvious that WSSV DNA accumulated in the nucleus of *Pm*VRP15-knockdown hemocytes.

### Expression and localization of *Pm*VRP15 in normal and *Pm*VRP15-knockdown hemocytes during WSSV infection

**Figure 7:**
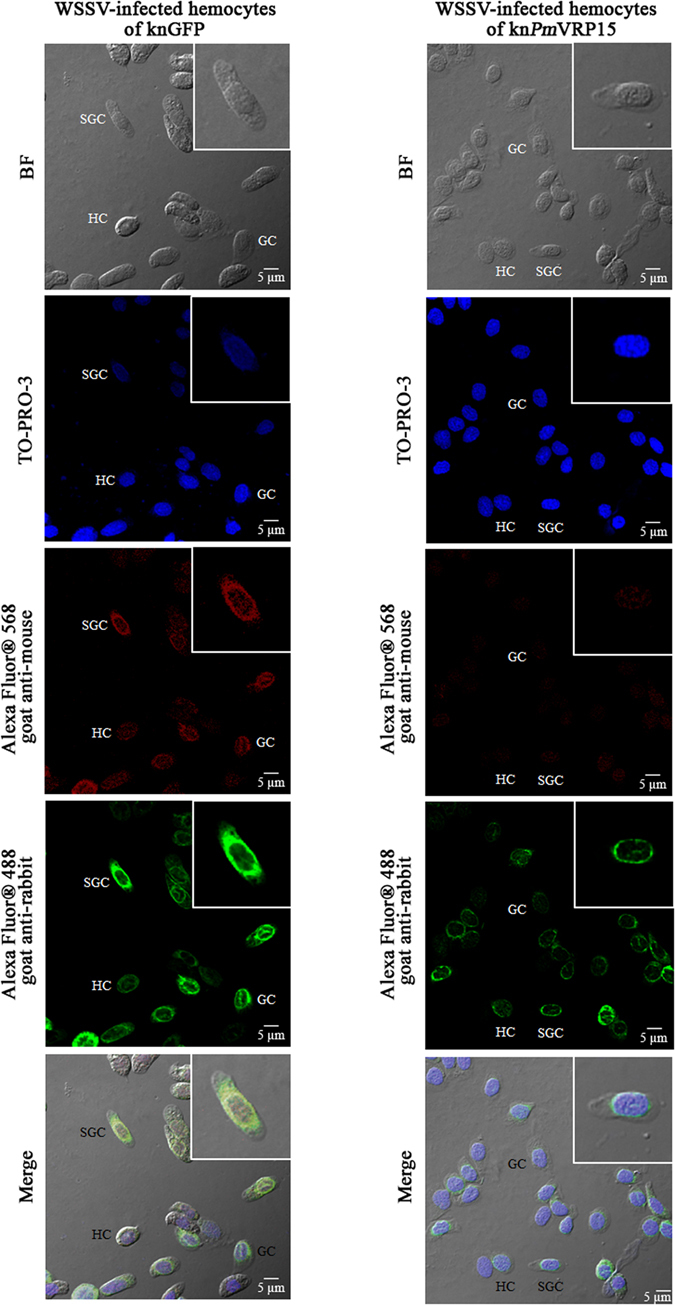
Immunofluorescent staining analysis of *Pm*VRP15 and VP28 in the WSSV-infected *Pm*VRP15-silenced hemocytes by confocal laser scanning microscopy. Hemocytes from *Pm*VRP15-silenced and GFP-silenced shrimp, infected by WSSV were collected at 48 hpi and probed by anti-*Pm*VRP15 and anti-VP28 antibodies. *Pm*VRP15 and VP28 proteins were visualized by secondary antibodies conjugated with Alexa Fluor^®^ 488 (green) and Alexa Fluor^®^ 568 (red), respectively. Nuclei were stained in blue. The GC, SGC and HC are granular, semigranular and hyaline cells, respectively. BFs are bright field images.

*Pm*VRP15 and VP28 in *Pm*VRP15 dsRNA and GFP dsRNA- treated hemocytes at 48 h post-WSSV infection was examined by confocal laser scanning microscopy using the antibodies specific to *Pm*VRP15 and VP28 coupled with different fluorescence-conjugated secondary antibodies. As shown in Fig. 7, *Pm*VRP15 (in green color) was observed in all three types of hemocytes, including granular cells (GC) semi-granular cells (SGC) and hyaline cells (HC). It also showed that *Pm*VRP15 protein was located around the edge of nucleus. The green fluorescent signal of *Pm*VRP15 was still observed in kn*Pm*VRP15 hemocytes; however, the intensity of the signal was much lower than that observed in the control (knGFP hemocytes). This suggested that *Pm*VRP15 was not completely silenced at 48 hpi, the time point at which *Pm*VRP15 was expressed at the highest level (Fig. 5b). Interestingly, hemocytes of kn*Pm*VRP15 showed significantly lower amounts of VP28 than those in knGFP cells. This indicated that knockdown of *Pm*VRP15 resulted in a reduction of VP28 protein.

## Discussion

This work aims to characterize the molecular properties of *Pm*VRP15 and investigate its role during WSSV infection. r*Pm*VRP15 was successfully expressed and purified. It is worth noting that r*Pm*VRP15 may be a toxic protein or very unstable since r*Pm*VRP15 was expressed within 1–2 h after IPTG induction only (data not shown). In general, the expression of hydrophobic heterologous proteins (usually membrane proteins) could be toxic to *E*. *coli*. A previous prediction based on amino acid sequence suggested that r*Pm*VRP15 could be a membrane protein with a single transmembrane helix^20^. As a result, *E*. *coli* C43 (DE3), which is effective in expressing toxic proteins, was used to express r*Pm*VRP15. In this research, n-decyl-ß-D-maltopyranoside (DM) was used in the purification process. Several studies have shown that DM does not interfere with the bioactivities of target proteins, denature or inactivate target proteins^24^. In nature, proteins bound to cell membranes have hydrophobic sites embedded inside the phospholipid bilayers and hydrophilic sites facing toward the water layer. DM can interact with the hydrophobic sites of proteins, providing a lipid-like environment.

The molecular mass of purified r*Pm*VRP15 determined by MALDI-TOF MS (15,899.9 Da) was in a good agreement with the calculated molecular mass. SEC was performed to monitor protein aggregation. Clearly, r*Pm*VRP15 required detergent to enhance protein solubility. AUC analysis demonstrated that, at 60 μM, r*Pm*VRP15 exists in three forms. The major peak was found at 172 kDa, indicating that most of r*Pm*VRP15 molecules existed in a tetrameric (or octameric) form. The CD spectrum of r*Pm*VRP15 revealed that r*Pm*VRP15 possessed a high content of α-helix, which is consistent with a result of our prediction of the secondary structure elements using a Web Server (data not shown).

Immunofluorescence confocal microscopy showed that *Pm*VRP15 protein was found around the edge of nucleus. A subcellular fractionation study revealed that *Pm*VRP15 was present in heavy membrane (plasma membrane and rough ER) and light membrane (polysomes, golgi apparatus, smooth ER) fractions. A previous study showed that *Pm*ERP15 (or *Pm*VRP15 in this study) was colocalized with an ER enzyme^21^. Taken together, this work confirmed that *Pm*VRP15 is likely to be a membrane-bound protein, which localized at the ER.

Leu *et al*.^21^ suggested that *Pm*ERP15 expression was induced due to ER stress exposure^21^. In addition, *Pm*ERP15 silencing increases the mortality of *P*. *monodon* after WSSV infection. As a result, *Pm*ERP15 was proposed to function in relieving ER stress, and in this regard, the absence of the protein led to the death of the shrimp due to the ER stress caused by WSSV infection. In contrast, Vatanavicharn *et al*., 2004 demonstrated that silencing of *Pm*VRP15 reduced the cumulative mortality rate of WSSV-infected shrimps^20^. To reconcile these conflicting data, a further study on role of *Pm*VRP15 during WSSV infection was carried out *in vitro*.

In primary hemocyte cultures, *Pm*VRP15 was up-regulated from 12 h onwards after WSSV infection. The highest level of *Pm*VRP15 expression in primary hemocyte cell cultures was observed at 48 h after WSSV infection, which was in agreement with the *in vivo* study^20^. At 48 hpi, *Pm*VRP15 was increased by 6.7-fold, in comparison to non-infected cells. This suggested that *Pm*VRP15 was induced by WSSV infection.

*In vitro Pm*VRP15-silencing did not affect WSSV copy number in whole hemocyte cells at 12 and 20 hpi. This suggested that *Pm*VRP15 plays no role in WSSV replication. This result was consistent with previously reported *Pm*VRP15 knockdown *in vivo* ^21, 22^. Interestingly, *Pm*VRP15 knockdown resulted in a significant increase in WSSV copy number ratio in nuclear and cytoplasmic fractions at 12 and 20 hpi. This suggested that silencing of *Pm*VRP15 caused an accumulation of WSSV DNA in nucleus. It was previously reported that WSSV DNA started to be synthesized from 6 hpi and the progeny WSSV were released from 12 hpi and peaked at 18 hpi^25^. It is possible that *Pm*VRP15 plays a role in viral exit from the nucleus, and that when *Pm*VRP15 was absent, the newly synthesized viral genomes accumulated in the nucleus.

Immunofluorescence microscopy showed that *Pm*VRP15 was observed in all three types of hemocytes, including GC, SGC and HC. However, the signal of *Pm*VRP15 in HC was much weaker than those in GC and SGC. This corresponds with the fact that WSSV selectively targets GCs and SGCs^26^. Although *Pm*VRP15-silencing showed that knVRP15 hemocytes contained similar amount of WSSV copy number to those in knGFP hemocytes, it appeared that, by confocal laser scanning microscopy, *Pm*VRP15 knockdown hemocytes had much lower level of VP28 envelope protein than those in knGFP group. This implied that *Pm*VRP15 most likely functions to accommodate viral assembly but not viral replication. Previously, *Pm*VRP15 was reported to interact with WSV399 viral tegument protein, which functions in capsid transport during viral trafficking and assembly^22^.

It is evident that several virus families, including both enveloped and nonenveloped DNA and RNA viruses, rearrange the ER-membrane to facilitate viral entry, replication and assembly^27–30^. For example, rotavirus uses ER as the final assembly site, where a viral intermediate, composed of the core shell and the middle layer capsid, migrates into the ER lumen to acquire the final capsid assembly.

**Figure 8:**
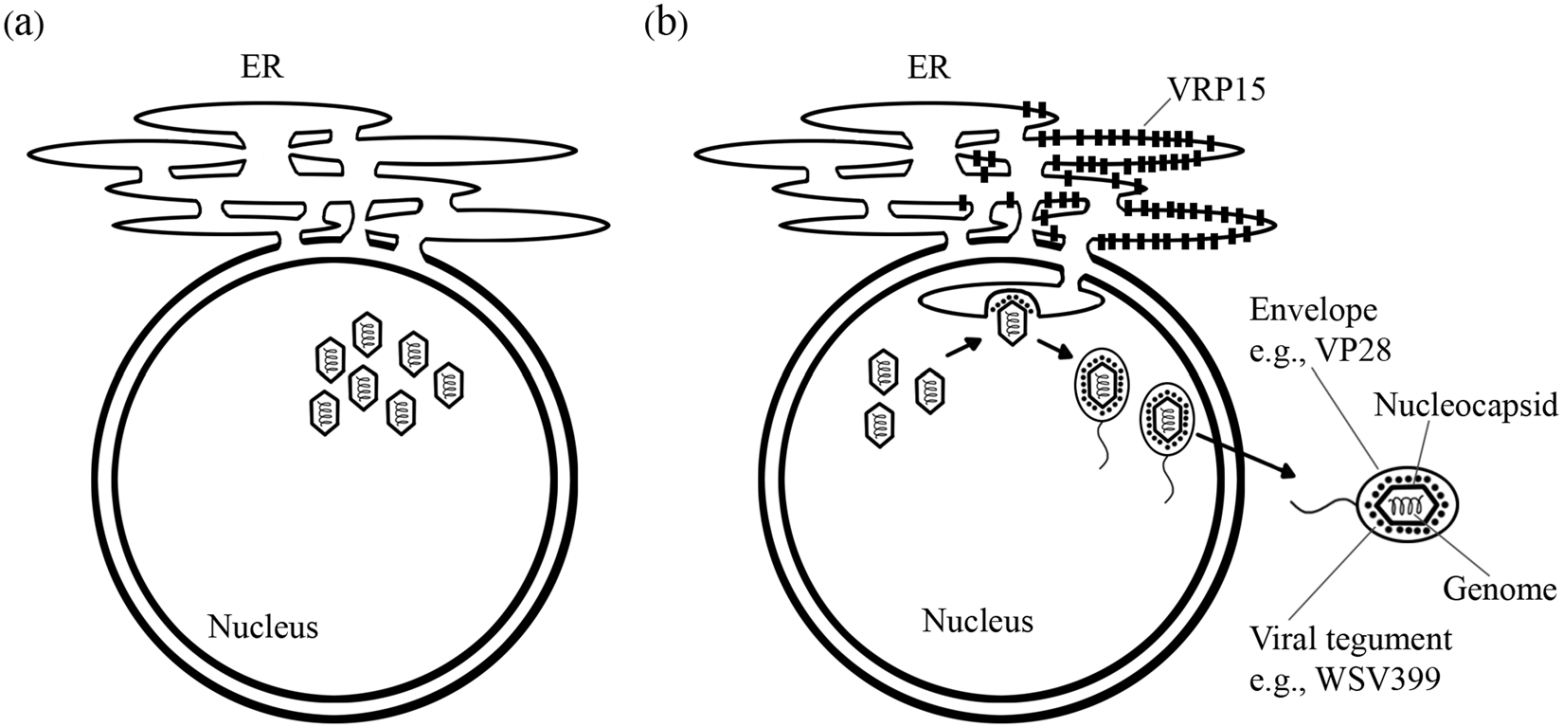
Proposed role of *Pm*VRP15 during WSSV infection. (**a**) In the absence of *Pm*VRP15, replicated WSSV genomes accumulate in the nucleus. (**b**) Overexpressed of *Pm*VRP15 at ER facilitates viral nuclear exit and assembly.

It is also common for viruses to use the host machinery for their advantages. For example, WSSV enters crayfish hematopoietic cells via multiple endocytic routes, including clathrin-mediated endocytosis, macropinocytosis and caveolae-mediated endocytosis^31^. In addition, *Pm*Rab7 is used in sorting and endocytic trafficking of WSSV in the host cells^32^. *P*. *monodon* Krupple-like factor (*Pm*KLFF) is also crucial for WSSV replication by enhancing WSSV immediate-early gene expression^33^. Previously, Li *et al*.^25^ proposed that the virus enters the cell via endocytosis and viral particle disassembly begins in the early endosome. The free nucleocapsid then injects the viral genome via a nuclear pore within the nucleus. After the mRNAs of immediate-early genes are produced, they travel into the cytoplasm and are translated into proteins by free ribosomes. The immediate-early proteins activate early and late proteins. The WSSV major envelope protein VP28 is expressed in the rER and migrates to the inner nuclear membrane. The newly synthesized viral genomes are assembled with the virus capsids, forming nucleocapsids which are then bud with VP28. We hypothesized that WSSV hijacks *Pm*VRP15 to facilitate viral nuclear egress and a maturation step, which may take place at ER (Fig. 8).

In conclusion, this work has confirmed that *Pm*VRP15 is a membrane-bound protein and localized at the ER. To our knowledge, this work is the first report to show that *Pm*VRP15 could possibly form an oligomer. In addition, *Pm*VRP15-silecning causes accumulation of viral DNA inside the nucleus, suggesting that *Pm*VRP15 plays a role in viral exit from the nucleus.

## Methods

### Expression and purification of recombinant *Pm*VRP15

*Escherichia coli* C43 (DE3) harboring pET22b(+)-*Pm*VRP15 plasmid was grown in LB medium containing 100 µg/ml ampicillin at 37 °C. A final concentration of 1 mM isopropyl-β-D-thio-galactoside (IPTG) was added and the cells were harvested and resuspended in 50 mM Tris-HCl buffer, pH 7.0 containing a Complete protease inhibitor cocktail tablet (Roche). Cells were disrupted by sonication and the supernatant was collected by centrifugation at 8,000 × *g*, 4 °C for 20 min. Membrane fraction was obtained by centrifugation at 100,000 × *g*, 4 °C for 1 h and homogenized in 50 mM Tris-HCl buffer, pH 7.0.

Membrane-bound proteins were solubilized in solubilizing buffer (50 mM Tris-HCl, pH 7, 20 mM Imidazole, 300 mM NaCl, 20% glycerol and 1% n-decyl-ß-d-maltopyranoside, DM) at 4 °C overnight. Recombinant *Pm*VRP15 (r*Pm*VRP15) was purified using Ni-NTA Sepharose^TM^ 6 Fast Flow (GE Healthcare). Crude proteins were incubated with Ni-NTA Sepharose^TM^ 6 FF bead in binding buffer (50 mM Tris-HCl, pH 7, 20 mM Imidazole, 10% glycerol and 0.1% DM), and non-specific proteins were removed by wash buffer (50 mM Tris-HCl, pH 7, 50 mM Imidazole, 5% glycerol and 0.1% DM). Recombinant *Pm*VRP15 protein was eluted with elution buffer (50 mM Tris-HCl, pH 7.0, 300 mM Imidazole, 5% glycerol and 0.1% DM) and analyzed by 15% sodium dodecyl sulphate polyacrylamide gel electrophoresis (SDS-PAGE).

Recombinant *Pm*VRP15 was further purified by HiTrap SP Fast Flow (GE Healthcare) and eluted in 20 mM MES, pH 6.5, 150 mM NaCl, 5% glycerol and 0.1% DM. The concentration of purified r*Pm*VRP15 was determined by Pierce^®^ BCA Protein Assay Kit (Thermo scientific). Western blotting was performed to identify His-tagged *Pm*VRP15 using a primary anti-His antibody (PAB10339 Abnova).

### MALDI-TOF Mass spectrometry (MALDI-TOF MS)

Mass spectrometric analysis was carried out on a Voyager DE-Pro MALDI-TOF MS instrument (Applied Biosystems) in linear positive mode. After time-delayed extraction, the ions were accelerated to 20 kV for TOF mass spectrometric analysis. A total of 400 laser shots were acquired and signal averaged per mass spectrum.

### Circular Dichroism (CD) spectroscopy

Secondary structure of r*Pm*VRP15 was estimated by circular dichroism (CD) spectroscopy using JASCO J-715 CD Spectropolarimeter with temperature controller and a 1 mm optical cuvette. The (+)-10-camphorsulfonic acid (CSA) was used as calibration standard before measuring CD spectrum. Spectra were recorded between 180–320 nm using a bandwidth of 2 nm and a response time of 2 sec with 50 mm/min scanning speed. Each spectrum was the average of three scans and background was subtracted with the spectrum of the buffer solution (blank). The CD spectra of r*Pm*VRP15 (0.42 mg/ml) were analyzed via DichroWeb (http://dichroweb.cryst.bbk.ac.uk)^34, 35^. Calculated spectra were obtained using K2D^36^ CONTINLL^37, 38^ and CDSSTR^39–41^ algorithms and the SMP180 protein dataset was used as ref. 42.

### Analytical ultracentrifugation

Size distribution of r*Pm*VRP15 was analyzed by analytical ultracentrifugation (AUC). Sedimentation velocity experiments were performed on an Optima XL-A analytical ultracentrifuge (Beckman Instruments) with an An-60 Ti rotor. Purified r*Pm*VRP15 (60 μM) was dissolved in 10 mM Tris-HCl, pH 7.5, 100 mM NaCl and 0.03% n-dodecyl β-D maltoside (DDM). The sample and reference buffer were injected into each side of a double-sector centerpiece and centrifuged at 20,000 rpm (Max. RCF = 40,275× g) at 20 °C for 16.5 h. The spectrum was monitored continuously using a time interval of 600 s per scan. The dataset from these multiple scans at 280 nm at different time intervals were then fitted to a continuous c(s) distribution model using the SEDFIT program^43, 44^. The partial-specific volume of *Pm*VRP15 was calculated to be 0.73 ml/g, based on the solvent density of 1.035 g/ml and the viscosity of 1.83 cP

### Size-exclusion chromatography

Purified r*Pm*VRP15 was loaded onto Superdex 200 column (GE Healthcare), pre-equilibrated with a running buffer (10 mM Tris-HCl, pH 7.5, 150 mM NaCl and 0.087% DM). *Pm*VRP15 was eluted using a running buffer. The column was calibrated using a protein standard mix (Bio-RAD) of thyroglobin (670 kDa), γ-globulin (158 kDa), ovalbumin (44 kDa), myoglobin (17 kDa) and vitamin B12 (1,350 Da). The calibration data fit to a straight line with r^2^ > 0.999.

### Shrimp

Juvenile black tiger shrimp, *P*. *monodon*, of about 10–15 g bodyweight were obtained from a shrimp farm in Nakhon Si Thammarat Province, Thailand. The animals were acclimated in laboratory tanks at ambient temperature (28 ± 4 °C), and maintained in aerated water with a salinity of 20 ppt for at least 7 days before use.

### Preparation of WSSV stock

The gill tissue from WSSV-infected moribund shrimp was collected for purification of viral particles. The purification method was slightly modified from Xie, X. *et al*.^45^. Gill tissue was homogenized in TNE buffer (50 mM Tris-HCl pH 8.5, 400 mM NaCl and 5 mM EDTA) and, then, centrifuged at 3,500 × *g* for 15 min at 4 °C. The supernatant was filtrated with MILLEX^®^-HP Filter Unit 0.45 μm (Merck Millipore) and centrifuged at 30,000 × *g* for 30 min at 4 °C to pellet the virions. The pellet was rinsed with TM buffer (50 mM Tris-HCl pH 7.5 and 10 mM MgCl_2_) and centrifuged at 3,500 × *g* for 10 min at 4 °C. The upper loose pellet was removed and the lower pellet resuspended in TM buffer, then centrifuged at 30,000 × *g* for 30 min at 4 °C. The pellet was suspended in TM buffer, aliquoted and stored at −80 °C until use.

### Primary Shrimp hemocyte cultures

Hemolymph was drawn from each shrimp using a sterile 1 ml syringe with 500 µl of anti-coagulant solution, pH 5.6 (0.82% (w/v) sodium chloride, 0.55% (w/v) citric acid, 1.98% (w/v) glucose and 0.88% (w/v) sodium citrate). The hemolymph-anticoagulant mixture was immediately centrifuged at 800 × *g* for 10 min at 4 °C to separate the hemocytes from the plasma. The hemocyte pellet was resuspended in 1 ml of L-15 culture medium (2x Leibovitz L-15 medium (Gibco) supplemented with 20% (v/v) fetal bovine serum (FBS), 1% (w/v) glucose, 0.4% (w/v) sodium chloride, 100 IU/ml penicillin and 100 µg/ml streptomycin; pH 7.6; adjusting the osmotic pressure to 750 ± 15 mOsm/kg with sodium chloride solution).

The number of hemocytes in the cell suspension was counted by a hematocytometer. Cell concentration was adjusted to 10^6^ cells per ml per well by L-15 fresh medium to obtain a final volume of 400 µl per well. The 24-well culture plate was incubated at 27 °C for 24 h, prior to being used in experiments.

### Subcellular fractionation

The hemocyte cell cultures (4 × 10^5^ cells per well) were incubated with 50 µl of the diluted WSSV solution (~15,000 viral copies/µl) and incubated at 27 °C. Subcellular fractionation by differential centrifugation was carried out as described in Taha, *et al*.^46^ with some modifications^46^. At 12 and 20 h post-WSSV infection, the hemocytes were resuspended with lysis buffer containing 10 mM Tris-HCl, pH 7.4, 10 mM NaCl, 0.5 mM MgCl_2_ and EDTA-free protease inhibitor cocktail (Roche), and cells were counted by hematocytometer. The hemocyte cells were homogenized and centrifuged at 1,200 × *g* for 5 min. Pellets were resuspended in 250 mM sucrose solution containing 10 mM MgCl_2_ and applied through an 880 mM sucrose cushion containing 0.5 mM MgCl_2_ by centrifugation at 1,200 × *g* for 10 min, to obtain the intact nuclei fraction. Meanwhile, supernatants of total cell lysate were re-centrifuged at 1,200 × *g* for 5 min and the cytosolic fraction (supernatant) was collected. The cytosolic fractions were further centrifuged at 16,000 × *g* for 10 min to isolate the heavy membrane pellet and the post-nuclear supernatant fraction, containing cytoplasm and light membrane. The post-nuclear fractions were then centrifuged at 100,000 × *g* for 120 min, in order to separate the light membrane pellet from the cytoplasmic supernatant. The total protein concentration of each fraction (intact nuclei, heavy membrane, light membrane and cytoplasm) was determined by BCA protein assay and 10 µg of proteins were loaded onto 15% SDS-PAGE for Western blot analysis. *Pm*VRP15 was probed by purified rabbit anti-*Pm*VRP15 polyclonal IgG antibody diluted 1:1000^20^. Anti-cyclophilin A, anti-PDI and anti-Nuclear Pore Complex (NPC) antibodies were used as a subcellular marker for cytoplasm, heavy membrane and intact nuclei, respectively. Anti-cyclophilin A antibody is a gift from Dr. Sirikwan Ponprateep, Srinakharinwirot University.

### *Pm*VRP15 mRNA expression in shrimp primary hemocyte culture in response to WSSV

A 400 µl of fresh L-15 culture medium, with or without 50 µl of the diluted WSSV solution (~15,000 viral copies/µl), was added to shrimp primary hemocyte culture. Total RNA was extracted from the hemocytes at 0, 6, 12, 24, 48 and 72 h post-WSSV infection using the TRI Reagent^®^ (Molecular Research Center), followed by DNase I, RNase-free (Thermo scientific) treatment. The total RNA was used for single-stranded cDNA synthesis by the First Strand cDNA Synthesis Kit (Thermo Scientific). The transcription level of target genes was identified by RT-PCR using an equal amount of cDNA template with gene-specific primers (See Supplementary Information, Table S1). Elongation factor-1 alpha (EF-1α) was used as an internal control. The PCR reaction was started with 94 °C for 3 min, followed by 33 cycles (for *Pm*VRP15) or 28 cycles (for EF-1α) of 95 °C for 30 sec, 58 °C for 30 sec and 72 °C for 30 sec, and a final extension at 72 °C for 5 min. PCR products were analyzed by 1.5% (w/v) agarose gel electrophoresis and the differential expression level of *Pm*VRP15 was reported as relative to EF-1α.

The expression levels of *Pm*VRP15 and EF-1α gene were further analyzed by quantitative real time RT-PCR (qRT-PCR). *Pm*VRP15 and EF-1α specific primers for qRT-PCR are shown in Supplementary Information, Table S1. The qRT-PCR was performed using the CFX96™ Real-Time PCR Detection System (Bio-Rad). PCR conditions were carried out as follows: 95 °C for 30 sec, followed by 40 cycles of 95 °C for 5 sec, 58 °C for 5 sec and 60 °C for 5 sec. The experiment was done in triplicate. The threshold cycle (*C*_T_) for each sample was analyzed by a mathematical model^47^. The data were shown as means ± standard deviations (SD). Statistical analysis was done using the one-way ANOVA followed by post hoc test (Duncan’s new multiple range test). Data differences were considered significant at *P < 0.05, **P < 0.01.

Comparative *C*_T_ method was used to compare the gene expression in two different samples. The fold change of gene expression was calculated using the following formula.

$$\begin{array}{rcl}{\rm{Fold}}\,{\rm{change}} & = & {2}^{-{\rm{\Delta }}{\rm{\Delta }}\mathrm{CT}}\\ {\rm{\Delta }}{\rm{\Delta }}{C}_{{\rm{T}}} & = & [({C}_{{\rm{T}}}\,{\rm{gene}}\,{\rm{of}}\,{\rm{interest}}-{C}_{{\rm{T}}}\,{\rm{internal}}\,{\rm{control}})\,{\rm{sample}}\,{\rm{A}}\\  &  & -\,({C}_{{\rm{T}}}\,{\rm{gene}}\,{\rm{ofinterest}}-{C}_{{\rm{T}}}\,{\rm{internal}}\,{\rm{control}})\,{\rm{sample}}\,{\rm{B}})]\end{array}$$

### Silencing of *Pm*VRP15 gene in shrimp primary hemocyte culture

*Pm*VRP15 dsRNA and GFP dsRNA (control) were synthesized using T7 RiboMAX™ Express Large Scale RNA Production System (Promega). Two sets of primers specific to each *Pm*VRP15 and GFP were designed as shown in Supplementary Information, Table S1. One of the specific primer pairs contained the T7 promoter at the 5′ end. The two PCR products were separately amplified by those primer pairs with the following conditions: 94 °C for 3 min, 30 cycles of 94 °C for 30 sec, 57 °C for 30 sec and 72 °C for 30 sec, and then a final extension at 72 °C for 5 min. Subsequently, the two PCR product templates were used to produce two complementary single-stranded RNAs by T7 RiboMAX^*TM*^ Express Large Scale RNA Production System (Promega). RQ1 RNase-free DNase was added and incubated at 37 °C for 15 min to remove the DNA template. Single-stranded RNAs were then purified by phenol-chloroform extraction. Double-stranded RNAs were produced by mixing equal amounts of each of the complementary single-stranded RNAs, incubated at 70 °C for 10 min, and then slowly cooled down at room temperature. *Pm*VRP15 dsRNA and GFP dsRNA were analyzed by 1% agarose gel electrophoresis and concentrations of both dsRNAs were determined by measuring absorbance at 260 nm.

The hemocyte cell cultures were divided into 3 groups and incubated with either 50 µl of L-15 medium (Group 1) or 50 µl of L-15 medium with 20 µg/well of GFP dsRNA (Group 2) or 50 µl of L-15 medium with 20 µg/well of *Pm*VRP15 dsRNA (Group 3). After 12 h, 50 µl of L-15 medium with or without, GFP dsRNA (10 µg/well) and *Pm*VRP15 dsRNA (10 µg/well) were added along with 50 µl of diluted WSSV solution (~15,000 viral copies/µl) and incubated at 27 °C. At 24 h post-WSSV infection, hemocyte cells were harvested and total RNA was extracted from hemocytes using the TRI Reagent^®^ (Molecular Research Center) followed by DNase I, RNase-free (Thermo scientific) treatment. Then, single-stranded cDNA was synthesized with the First Strand cDNA Synthesis Kit (Thermo Scientific) and used for analysis of *Pm*VRP15 mRNA expression by RT-PCR with EF-1α as an internal reference. The PCR product was analyzed by 2% (w/v) agarose gel electrophoresis to confirm the silencing of *Pm*VRP15 transcripts.

### Quantification of WSSV copy number in whole cells and in the nuclear and cytoplasmic fractions of normal and *Pm*VRP15 gene-silenced hemocytes after WSSV infection

The hemocyte cell cultures were divided into 3 groups and incubated with 50 µl of either L-15 medium, GFP dsRNA (20 µg/well) or *Pm*VRP15 dsRNA (20 µg/well). After 12 h, 50 µl of either L-15 medium, GFP dsRNA (10 µg/well) or *Pm*VRP15 dsRNA (10 µg/well) were added, along with 50 µl of the diluted WSSV solution (~15,000 viral copies/µl) and incubated at 27 °C. At 12 and 20 h post-WSSV infection, the hemocyte cells were collected and used as whole cells or cytoplasmic and nuclear fractions for quantification of WSSV copy numbers.

In brief, cytoplasmic and nuclear fractions were separated by differential centrifugation as shown in Fig. 1. The hemocyte cells were homogenized and centrifuged at 1,200 × *g* for 5 min. Pellets were resuspended, layered onto a sucrose cushion and centrifuged at 1,200 × *g* for 10 min, to obtain the nuclear fraction. Supernatants of total cell lysate were re-centrifuged at 1,200 × *g* for 5 min to collect the cytoplasmic fraction (supernatant). To confirm that there was no cross-contamination between the nuclear and cytoplasmic fractions, 10 µg of proteins from each fraction were applied on SDS-PAGE. Anti-NPC antibody and anti-cyclophilin A antibodies were used as a subcellular markers for the nuclear and cytoplasmic fractions, respectively. Pierce^TM^ Fast Western Blot Kit and SuperSignal^TM^ West Femto were then used for Western blot detection.

The total DNA was extracted from either whole cells or the nuclear and cytoplasmic fractions. WSSV copy number was then determined by Real-time PCR using WSSV1011F/WSSV1079R primers (See Supplementary Information, Table S1). The experiment was carried out in triplicate and WSSV recombinant plasmid (known copy number) was used as the standard for quantification^48^.

### Visualization of normal and *Pm*VRP15-knockdown hemocyte-infected by WSSV using confocal immunofluorescence microscopy

Shrimp (~10 g body weight) were injected with 25 µl of 150 mM sodium chloride containing either GFP dsRNA (10 µg/g shrimp) or *Pm*VRP15 dsRNA (10 µg/g shrimp). After 24 h, the shrimp were repeatedly injected with either GFP dsRNA (5 µg/g shrimp) or *Pm*VRP15 dsRNA (5 µg/g shrimp), together with 30 µl of the diluted WSSV solution (~4 × 10^3^ viral copies). At 48 h post-WSSV injection, hemolymph was immediately fixed with 4% (w/v) paraformaldehyde in PBS, pH 7.4 (ratio 1:1). Hemocytes were collected by centrifugation at 800 × *g* for 10 min, washed three times with PBS, pH 7.4, then immersed in PBS, pH 7.4, and kept at 4 °C until used.

Hemocytes (1 × 10^6^ cells/mL) were attached onto a microscope slide (Polysine slides, Thermo Scientific) and incubated with 0.1% (v/v) Triton-X 100 in PBS for 5 min. After rinsing the slide in PBS, it was immersed in a blocking solution (10% (v/v) FBS in PBS) for 1 h, followed by washing in PBS. The fixed hemocytes were incubated with a 1:500 dilution of purified rabbit anti-*Pm*VRP15 polyclonal antibody and 1:100 dilution of mouse anti-VP28 polyclonal antibody in PBS, pH 7.4, containing 1% (v/v) FBS. After PBS washing, the fixed hemocytes were incubated with a 1:500 dilution of Alexa Fluor^®^ 488 goat anti-rabbit IgG antibody (Invitrogen) and Alexa Fluor^®^ 568 goat anti-mouse IgG antibody (Invitrogen) in PBS, pH 7.4. The nucleus was then stained with a 1:1500 dilution of TO-PRO-3 iodide (Invitrogen) in PBS, pH 7.4. Coverslips were then coated with Prolong Gold Antifade Reagent (Invitrogen) and kept in the dark at 4 °C until they were visualized under a confocal laser scanning microscope (Olympus).

## Electronic supplementary material

Supplementary Information
