# Supplementary material for: Molecular Characterization of Viral Responsive Protein 15 and Its Possible Role in Nuclear Export of Virus in Black Tiger Shrimp Penaeus monodon
Source: Sci Rep. 2017 Jul 26;7:6523. doi: 10.1038/s41598-017-06653-7 (PMC5529560; doi:10.1038/s41598-017-06653-7)
Supplement: Supplementary file 1 — Supplementary Information [file 41598_2017_6653_MOESM1_ESM.pdf]

Molecular Characterization of Viral Responsive Protein 15 and Its Possible Role in Nuclear Export of Virus in Black Tiger Shrimp *Penaeus monodon*

Krisadaporn Jaturontakul[1‡], Thapanan Jatuyosporn[1‡], Pasunee Laohawutthichai[1], Sun-Yong Kim[2], Tomoyuki Mori[2], Premruethai Supungul[3], Toshio Hakoshima[2], Anchalee Tassanakajon[1] and Kuakarun Krusong[1*]

[1] Center of Excellence for Molecular Biology and Genomics of Shrimp, Department of Biochemistry, Faculty of Science, Chulalongkorn University, Bangkok 10330, Thailand

[2] Structural Biology Laboratory, Nara Institute of Science and Technology, 8916-5 Takayama, Ikoma, Nara 630-0192, Japan

[3] National Center for Genetic Engineering and Biotechnology (BIOTEC), National Science and Technology Development Agency (NSTDA), Pathumthani 12120, Thailand

[‡] Two authors contributed equally to this work

[*] To whom correspondence should be addressed:
Kuakarun Krusong, Ph.D.
Department of Biochemistry, Faculty of Science,
Chulalongkorn University, Bangkok 10330, Thailand
Tel: +66 (0)2 218 5413
Email: Kuakarun.K@chula.ac.th

Supplementary Information

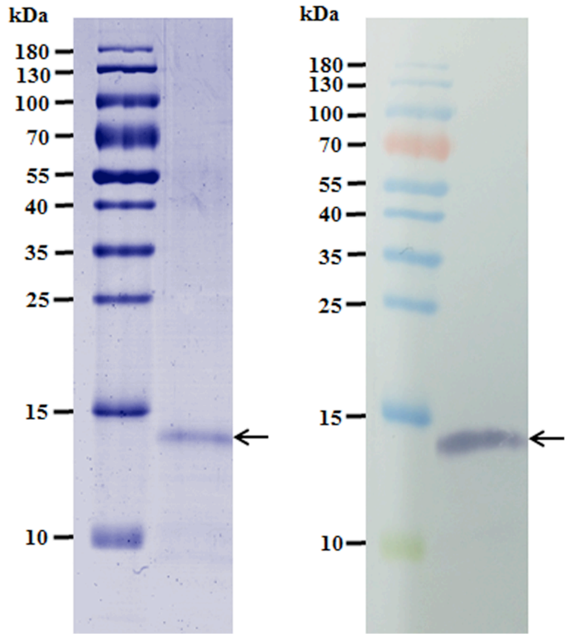

Fig. S1 15% SDS-PAGE of purified r*Pm*VRP15 by Coomassie brilliant blue staining (A) and Western blotting (B). The arrows indicate r*Pm*VRP15, which appeared just below 15 kDa, close to the estimated size of r*Pm*VRP15 (15.86 kDa).

Table S1 Nucleotide sequences of the primers

Primer	Sequence (5´-3´)	Usage
EF1-α QF	GGTGCTGGACAAGCTGAAGGC	qRT-PCR
EF1- α QR	CGTTCCGGTGATCATGTTCTTGATG	qRT-PCR
GFP-F	ATGGTGAGCAAGGGCGAGGA	dsRNA synthesis
GFP-R	AGAAGGAAGGGCGCTGAC	dsRNA synthesis
VP28-FRT	TCACTCTTTCGGTCGTGTCG	RT-PCR
VP28-RRT	CCACACACAAAGGTGCCAAC	RT-PCR
VP28QF	GGGAACATTCAAGGTGTGGA	qRT-PCR
VP28QR	GGTGAAGGAGGAGGTGTTGG	qRT-PCR
*Pm*VRP15-1F RNAi	GGATCCTAATACGACTCACTATAGGCGCGA CCGAGCCAAGAGAACAT	dsRNA synthesis
*Pm*VRP15-1R RNAi	TGAGCTGACGGAAGGCCACAGA	dsRNA synthesis
*Pm*VRP15-2F RNAi	CGCGACCGAGCCAAGAGAACAT	dsRNA synthesis
*Pm*VRP15-2R RNAi	GGATCCTAATACGACTCAC TATAGGTGAGCTGACGGAAGGCCACAGA	dsRNA synthesis
*Pm*VRP15-QF	CGTCCTTCAGTGCGCTTCCATA	qRT-PCR
*Pm*VRP15-QR	ACAGCGACTCCAAGGTCTACGA	qRT-PCR
*Pm*VRP15-RTF	CGATCACCACTCTCGTTCTT	RT-PCR
*Pm*VRP15-RTR	ACAGCGACTCCAAGGTCTACGA	RT-PCR
WSSV1011F	TGGTCCCGTCCTCATCTCAG	WSSV copy number detection
WSSV1079R	GCTGCCTTGCCGGAAATTA	WSSV copy number detection

Full-length blots

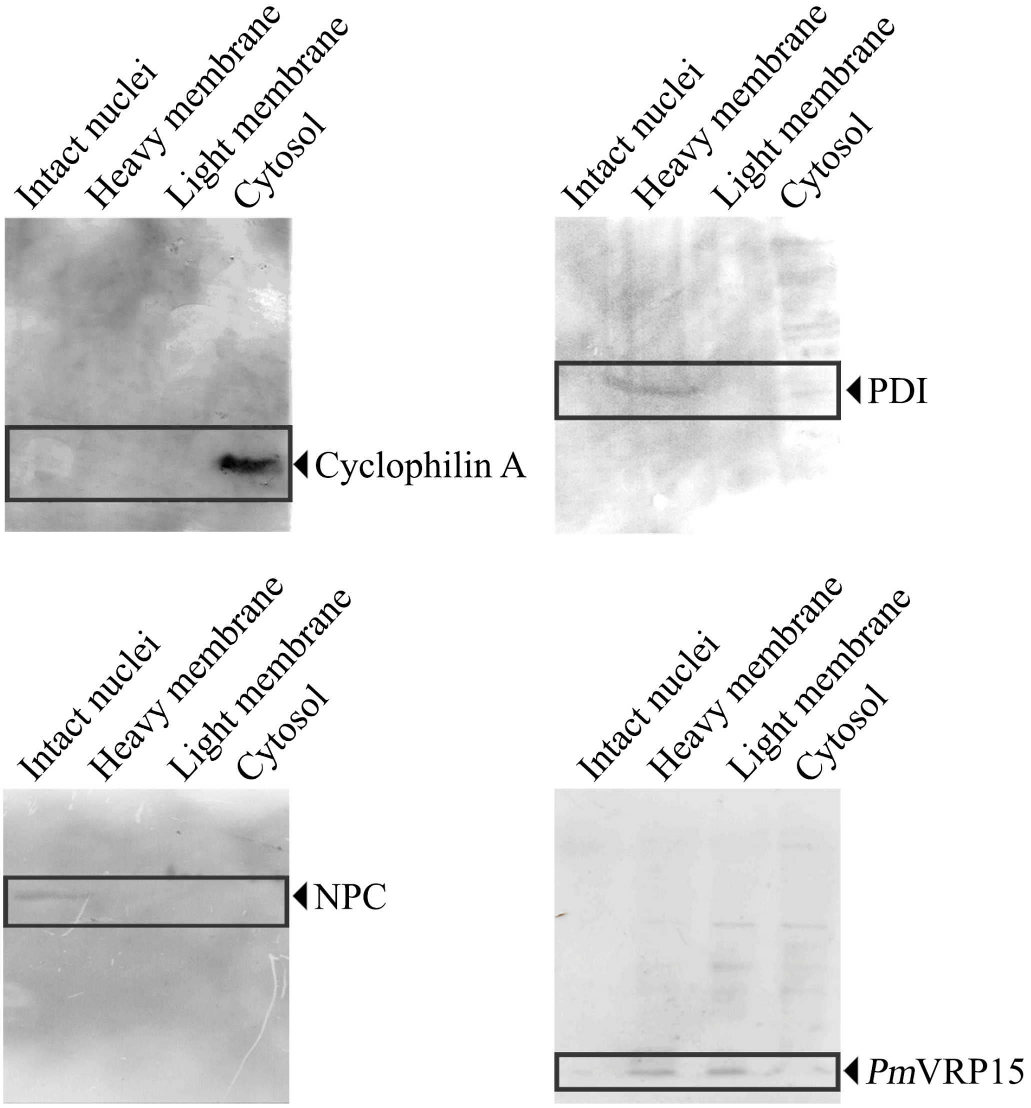

Fig. 4

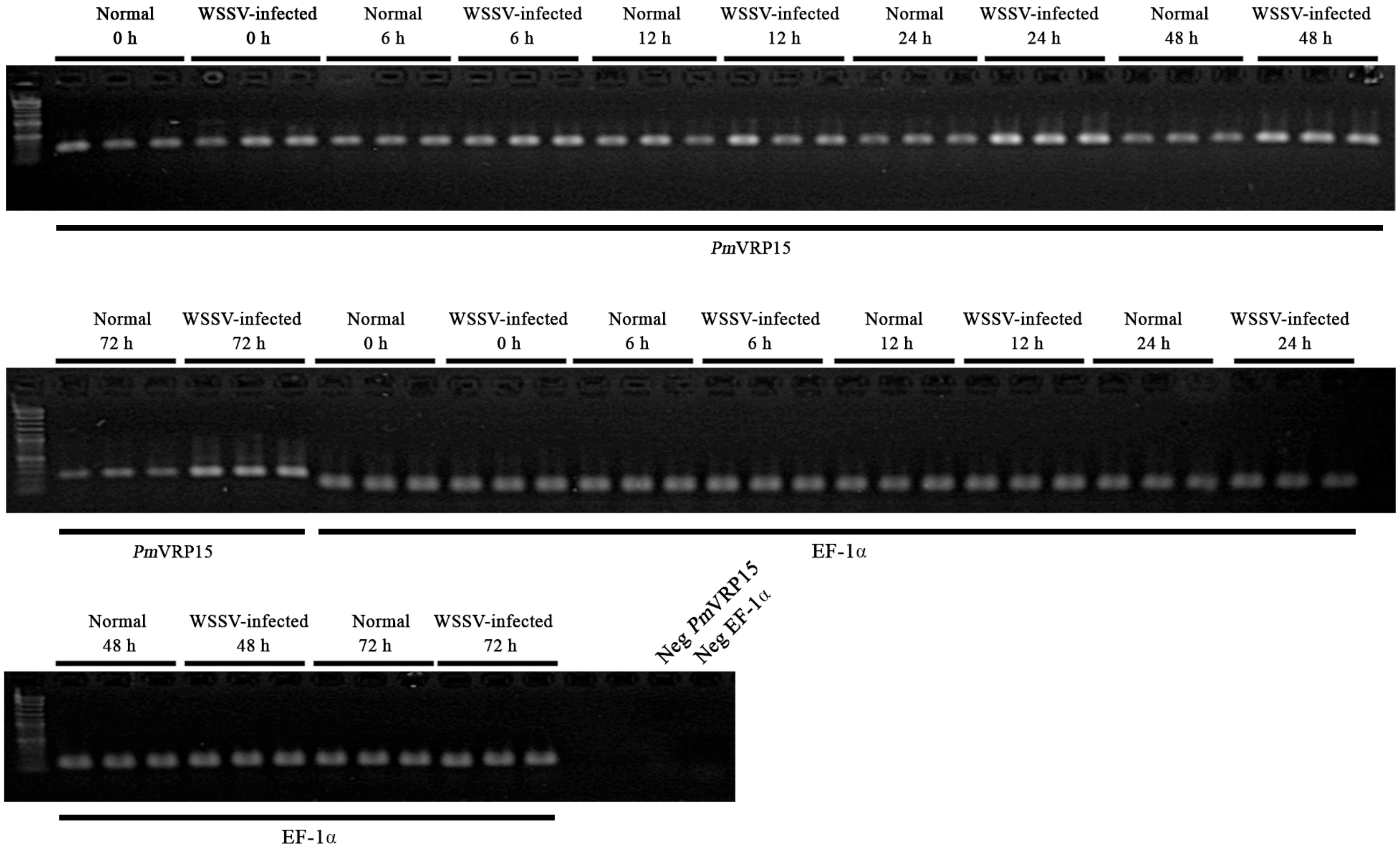
Normal 0 h
WSSV-infected 0 h
Normal 6 h
WSSV-infected 6 h
Normal 12 h
WSSV-infected 12 h
Normal 24 h
WSSV-infected 24 h
Normal 48 h
WSSV-infected 48 h
PmVRP15
Normal 72 h
WSSV-infected 72 h
Normal 0 h
WSSV-infected 0 h
Normal 6 h
WSSV-infected 6 h
Normal 12 h
WSSV-infected 12 h
Normal 24 h
WSSV-infected 24 h
PmVRP15
EF-1α
Normal 48 h
WSSV-infected 48 h
Normal 72 h
WSSV-infected 72 h
Neg PmVRP15
Neg EF-1α
EF-1α

Fig. 5

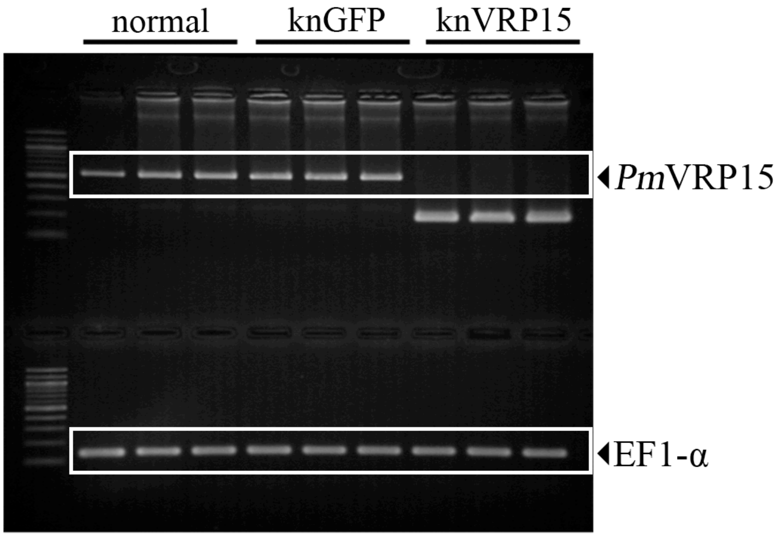
normal
knGFP
knVRP15
PmVRP15
EF1-α

Fig. 6a

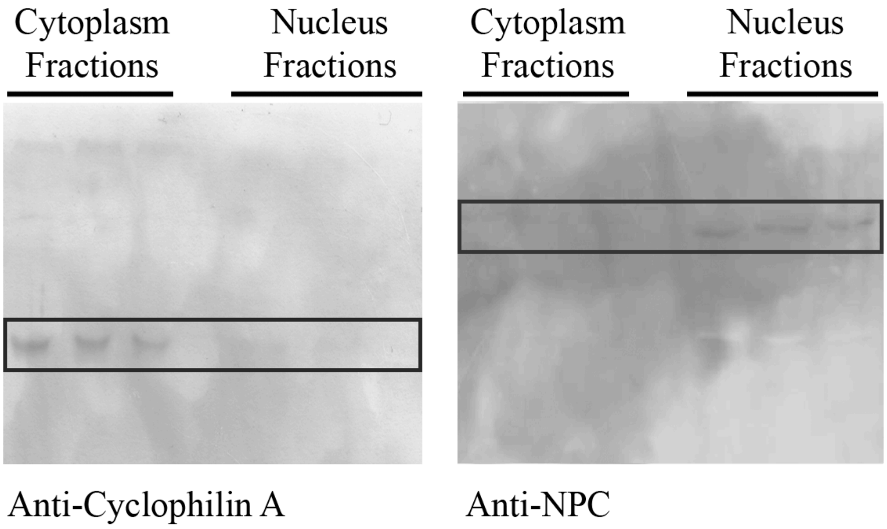
Cytoplasm Fractions
Nucleus Fractions
Cytoplasm Fractions
Nucleus Fractions
Anti-Cyclophilin A
Anti-NPC

Fig. 6c
